# Mapping Core Communication Networks in Bacteria

**DOI:** 10.1371/journal.pbio.0030359

**Published:** 2005-09-27

Single-celled bacteria may appear simple by some standards, but these tiny cells employ sophisticated systems for processing stimuli. One especially important class of signaling molecules that help bacteria coordinate the activities of daily life is called the two-component signal transduction system. This system—comprised of enzymes called histidine kinases and their target molecules, the response regulators—allows bacteria to sense and respond to their surroundings by transforming various environmental cues, such as sugars, peptides, and antibiotics, into physiological responses. These environmental signals trigger a chemical reaction in histidine kinases called autophosphorylation, in which the kinase transfers a phosphoryl group from a molecule of ATP (which powers many cellular processes) to one of its own amino acids. The enzyme then transfers the phosphoryl group to its target response regulator, producing changes in gene expression, motility, protein breakdown, and various other cellular processes.

Though it's possible to identify histidine kinases and response regulators in bacteria by analyzing their genome sequences, it's far more difficult to determine how the two components interact: do they form monogamous pairs or behave more promiscuously? To characterize the range of possible interactions and the intracellular changes they bring about, Michael Laub and colleagues developed a novel method of mapping the connections between histidine kinases and response regulators in the freshwater bacterium *Caulobacter crescentus*. By combining genetic and biochemical analyses on a system-wide scale, the authors rapidly identified two-component signaling pathways in *C. crescentus*, including pathways required for core cell processes.[Fig pbio-0030359-g001]

**Figure pbio-0030359-g001:**
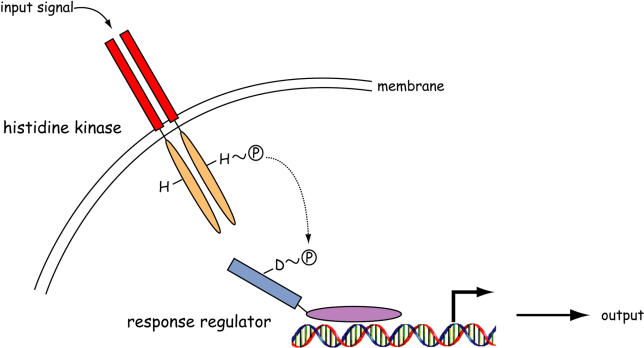
In the two-component signaling pathway, a stimulus causes a histidine kinase to autophosphorylate on a conserved histidine (H) residue. The phosphoryl group (P) is then transferred to a conserved aspartate (D) residue of a cognate response regulator; when the response regular phosphorylates, it triggers a physiological response, such as gene transcription

Laub and colleagues first identified 106 two-component signal transduction genes (62 histidine kinases and 44 response regulators) from the genome sequence. Then they created mutant strains of bacteria that each had one of the 106 genes deleted (called deletion strains) to learn how the genes function. The phenotypes, or physical characteristics, of the mutant strains allowed the scientists to identify 39 genes required for cell cycle progression, growth, and morphogenesis, including nine genes essential to survival. To address the promiscuity question and figure out the likely phosphotransfer pairings among the components, the authors developed a biochemical method, called phosphotransfer profiling, that quickly identifies a histidine kinase target by tracking the transfer of radioactive (called radio-labeled) phosphates from the kinase to the target.

The authors validated their in vitro technique on two-component proteins from *E. coli* (a system in which many of the living bacteria's kinase-target pairings are known) by showing that the kinases preferentially phosphorylated their known targets in the test tube as well, forming promiscuous unions only after prolonged periods. Confident that their method would also work for other bacteria, the researchers applied it to *C. crescentus* and determined the likely pairings for previously identified histidine kinases. Since the authors again observed a high preference for known targets, they were confident that these pairings were real, and the method could be used to identify targets of other, uncharacterized kinases. Based on the deletion analysis results, Laub and colleagues focused on a histidine kinase that appears essential for growth or survival and identified a single target response regulator. Eliminating the expression of each gene produced nearly identical phenotypes, revealing the pair's role in a signaling pathway that maintains the integrity of the bacteria's cellular envelope. Because two-component systems aren't found in humans, this cellular-envelope pathway may prove an effective antibiotic target against pathogenic bacteria, based on the effectiveness of other antibacterial therapies aimed at the cell membrane.

The library of deletion strains the authors developed will be a valuable resource, not only for identifying other two-component pathways in *C. crescentus* but also for studying aspects of the cell cycle and development, thanks to the bacterium's unusual life cycle—it divides asymmetrically, producing a motile daughter cell and a stationary one that can then differentiate into the motile version. And since the techniques presented here should work in any organism with two-component signaling (most bacteria, fungi, and many plants), researchers can apply them to the tall task of decoding the labyrinthine communication systems that sustain cellular life. —*Liza Gross*

